# Association of Brachial-Ankle pulse pressure with coronary artery stenosis severity: A sex-specific cross-sectional study in Chinese adults

**DOI:** 10.1371/journal.pone.0350269

**Published:** 2026-06-05

**Authors:** Junnan Song, Haiyan Wang, Yali Mao, Lin Zheng, Lili Chen, Chunhui Yang, Zhongsu Shi, Huan Liu, Annuo Liu

**Affiliations:** 1 School of Nursing Anhui Medical University, Hefei, China; 2 Department of Nursing, Anhui No.2 Provincial People’s Hospital, Hefei, China; 3 Department of Nursing, The First Affiliated Hospital of Anhui Medical University, Hefei, China; Johns Hopkins University School of Medicine, UNITED STATES OF AMERICA

## Abstract

**Objectives:**

To investigate the association between brachial and ankle pulse pressure and the severity of coronary artery stenosis, as well as to explore sex-specific differences in these associations.

**Methods:**

A total of 218 patients who attended a tertiary hospital in China from 1 July 2023–31 January 2024 were selected for the study. The brachial artery pulse pressure, ankle artery pulse pressure, degree of coronary artery stenosis, and the number of major vascular lesion branches were standardized and measured. Multiple logistic regression and receiver operating characteristic curves were employed for analysis.

**Results:**

Pulse pressure values at all four measurement sites (left and right brachial, left and right ankle) were significantly higher in the severe coronary stenosis group (T3) compared with the mild stenosis group (T1; all P < 0.001). Multivariate logistic regression analysis showed that left brachial pulse pressure (LPP) was independently associated with severe coronary stenosis (OR: 0.048, 95% CI: 0.010–0.227, P = 0.01). ROC analysis revealed that LPP had favorable predictive performance for severe coronary stenosis, with AUC values of 0.862 in men and 0.854 in women. Gender differences were observed in ankle pulse pressure: right ankle PP showed numerically higher AUC values in men (0.847), while left ankle PP showed numerically higher AUC values in women (0.842).

**Conclusion:**

Depending on gender and the patient’s specific condition, brachial and ankle pulse pressure are critical in assessing coronary stenosis. Site-specific pulse pressure measurements may help identify patients at higher risk of coronary stenosis, enabling effective interventions to better safeguard their cardiovascular health.

## Introduction

Coronary heart disease (CHD) continues to pose a significant global health challenge and is one of the leading causes of death worldwide [1]. About 7.4 million people die of CHD each year [2]. Its morbidity and mortality are on the rise globally [3]. This disease burden not only undermines the quality of life of patients but also poses a serious threat to their health and safety, accompanied by high costs associated with treatment and long-term care [4]. The severity of CHD is primarily determined by the degree of coronary artery stenosis, which is typically assessed by evaluating the extent of narrowing in the coronary lumen diameter [5]. Although prompt pharmacological therapy can temporarily control symptoms of luminal stenosis (stenosis < 50%), it may still progress to coronary heart disease over time without anti-atherosclerotic treatment [6]. Therefore, timely assessment and effective identification of coronary artery stenosis are crucial for guiding further therapeutic and nursing interventions.

Currently, invasive coronary angiography is considered the gold standard for detecting coronary artery stenosis; however, it is expensive and carries some risks [7]. Additionally, clinical risk scores utilize established biomarkers such as age, gender, smoking history, inflammation, and lipoproteins to identify individuals at high risk for coronary artery stenosis [8,9]. However, these scores often fail to detect a significant proportion of coronary artery disease cases, and just has good effectiveness among young adults [10] and women [11].

Recent studies have shown that pulse pressure (PP) is an independent risk factor for cardiac, cerebral, and renal vascular disease [12]. In patients with coronary artery disease, the effect of PP on the vascular system is related to output per beat and arterial wall elasticity, which reflects the flexibility of the conduit arteries [13,14]. Loss of arterial elasticity not only leads to elevated systolic blood pressure, increased cardiac load, and vascular remodeling [15], but also affects small peripheral arteries and microcirculation [16,17], ultimately increasing peripheral vascular resistance [18]. Therefore, PP is a good indicator for predicting the occurrence and prognosis of coronary stenosis events.

Additionally, previous studies have shown that PP has a higher predictive value than systolic blood pressure (SBP) and diastolic blood pressure (DBP) in assessing the risk of coronary artery stenosis due to the fact that PP increases when SBP is stable, in response to increased stiffness of the large arteries [19]. Whereas when both SBP and DBP are elevated, pulse pressure remains unchanged, which is due to an increase in peripheral vascular resistance [20]. PP has the advantages of being noninvasive, low-cost [21], easy to calculate, having a high predictive value, and being convenient [22] compared with other measurement methods.Hence, the development of a simple non-invasive screening tool for vascular disease that would be used by all health care personnel is of great importance.

Although several previous studies have examined the association between PP and the degree of coronary artery stenosis in patients with CHD [23,24], their main focus was on aortic PP rather than noninvasive extremity PP. Furthermore, although increased upper extremity PP is associated with an increased risk of CHD [25], most of these studies have focused on the brachial artery and have neglected the ankle artery. However, Ankle PP may have unique advantages in predicting coronary artery stenosis.The ankle PP can reflect the changes in arterial compliance reduction on the time diagram of incident pressure waves generated by left ventricular ejection and reflected waves produced by the arterial system [26]. Ankle PP not only reflects the changes in elasticity of the lower limb vessels but may also provide additional information on the risk of coronary artery stenosis through its indirect effects on the systemic arterial system [21]. Measurement of ankle PP is simple, noninvasive, and well-suited for widespread application in clinical nursing practice.Studies on the differences in the predictive effects of ankle PP on coronary artery stenosis are lacking. In clinical nursing practice in more Chinese hospitals, the focus of attention on pulse pressure is mainly on the brachial artery, with relatively little attention paid to the ankle artery. However, monitoring of ankle PP may offer a new perspective for the early identification and intervention of CHD. Therefore, this study also serves as a reminder for nurses to pay attention to changes in patients’ ankle artery pressure when monitoring their blood pressure.

In the context of nursing, understanding these differences can help nurses make more informed decisions regarding patient monitoring, early intervention, and individualized care plans. Therefore, the purpose of this study was: [1] to investigate the association between brachial and ankle pulse pressure and the severity of coronary artery stenosis; and [2] to explore the association between these pulse pressure measurements and the number of stenotic branches..

## Methods

### Design

This was a retrospective cross-sectional study aiming to evaluate the association between brachial and ankle pulse pressure and the severity of coronary artery stenosis.

### Participants

Data were accessed for research purposes on 13 February 2025 for 218 patients who had received treatment and undergone coronary angiography at a tertiary hospital in China between 1 July 2023 and 31 January 2024. The authors did not have access to information that could identify individual participants during or after data collection.

Inclusion Criteria: 1. Aged 18 years and older; 2. Completion of coronary angiography with complete angiographic information; 3. Completion of relevant laboratory tests.

Exclusion Criteria: 1. Patients with previous or newly diagnosed severe liver or kidney failure, malignant tumors, infectious diseases, etc.; 2. Patients with severe mental illness; 3. Patients with missing data on laboratory tests, blood pressure, and imaging results. 4.Preexisting peripheral vascular disease(PVD).Ultimately, 218 study subjects were included in the final analysis.

### Data collection

The general information was collected from the patients’ medical records, including age, gender, body mass index, smoking history, medical history, weekly exercise, sleep quality, presence of cohabiting individuals, and education level by trained researchers. Poor sleep quality is defined as sleeping less than 7–8 hours per night. Education levels are categorized as follows: elementary school and below, high school or junior college, and university and above.

Blood pressure measurements were performed prior to coronary angiography, with patients in a supine position after at least 10 minutes of rest. No patient reported active chest pain or angina symptoms at the time of blood pressure measurement. Two consecutive measurements were taken, with a two-minute interval between each. If the difference between the two systolic blood pressures (SBP) or diastolic blood pressures (DBP) in a given area exceeded 10 mmHg, a third measurement was conducted. The final result recorded was the average of the two measurements that were closest to each other. The pulse pressure (PP) was calculated as follows: pulse pressure (PP) = systolic blood pressure (SBP) - diastolic blood pressure (DBP).

All patients underwent percutaneous coronary angiography using the standard Judkins method, which includes a total of eight projection positions. The degree of stenosis in the coronary arteries and the number of major stenotic branches were assessed based on the angiogram results. The degree of stenosis in each patient’s coronary arteries was quantified using the Gensini score (GS) [27]. The GS is a tool for quantifying the degree of coronary artery stenosis. It weights each vessel or vessel segment according to the usual blood flow to the left ventricle and distinguishes between the right and left dominant coronary systems. When scoring, a multiplying factor is applied to each lesion based on its location in the coronary tree and the functional significance of the area it supplies. The GS takes into account both the degree of stenosis and the location of the lesion, as well as the cumulative effect of multiple lesions. Additionally, it considers the impact of collateral circulation: if a vessel segment is totally occluded or has 99% stenosis with collateral supply, a collateral adjustment factor is used, and the adjustment is reduced according to the extent of disease in the vessel providing the collaterals [27]. This makes the GS a relatively reliable numerical value that provides valuable information for prognostic assessment and helps predict the prevalence of coronary artery plaques. It is worth noting that while some assessments may ignore coronary artery plaques with stenosis less than 50%, these lesions are included in the GS system [27,28]. The GS is easy to calculate and apply, and the final score is the sum of all lesion scores. Calculated as follows: the score for each lesion = the score for the degree of stenosis of the vessel × the coefficient corresponding to the site of the lesion. The overall Gensini score for each patient was the sum of the scores from all affected vessels. The number of branches with significant coronary artery stenosis was classified based on the imaging results as follows: no stenosis (N0), single major stenosis (N1), or two or more major stenoses (N2).

### Ethical considerations

The investigation conforms to the principles outlined in the Declaration of Helsinki. All participants signed written informed consent forms before data collection.Ethical approval was obtained from the Ethics Committee of Anhui No.2 Provincial People’s Hospital ((R)2025−008).

### Data analysis

SPSS 27.0 and R 4.4.1 software were used for data analysis. Measurements conforming to a normal distribution were expressed as mean ± standard deviation ($\bar{x}$ ± s). Patients were categorized into T1 (Gensini≤3,n = 77), T2 (3 < Gensini<18,n = 68), and T3 (Gensini≥18,n = 73) groups according to the tertile of their Gensini score. The number of major coronary stenosis branches was categorized into no stenosis group (N0, n = 41), single major stenosis group (N1,n = 56), and two or more major stenosis groups (N2, n = 121). Comparisons between groups were analyzed using one-way ANOVA if the variance was uniform, or the Kruskal-Wallis test if the variance was not uniform. Measures with a non-normal distribution were expressed as median and interquartile range [M (P25-P75)], with comparisons between groups made using the Kruskal-Wallis test. Count data were expressed as frequency (%), with group comparisons conducted using the χ² test.

Pearson’s correlation and Spearman’s rank analysis were employed to assess the correlation between left brachial pulse pressure (LPP), right brachial pulse pressure (RPP), left ankle pulse pressure (LLPP), right ankle pulse pressure (RLPP), and the Gensini score. The association of each pulse pressure (PP) with categorical Gensini score tertiles (T1, T2, T3) and categorical number of stenotic branches (N0, N1, N2) was analyzed using multivariate logistic regression models, with T3 and N2 as reference groups, respectively. Results were expressed as odds ratios (ORs) and 95% confidence intervals (95% CIs). Model 1 was unadjusted for confounders; Model 2 was adjusted for age and sex; Model 3 was adjusted for BMI, smoking, creatinine (CREA), glucose (GLU), triglycerides (TG), cholesterol (CHOL), high-density lipoprotein(HDL), non-high-density lipoprotein(non-HDL), and D-dimer fibrin degradation product(D-Dimer) based on Model 2.

Restricted cubic spline (RCS) curves were utilized to determine whether there was a linear relationship between pulse pressure and coronary stenosis in the extremities. Additionally, the ability of each pulse pressure measure to discriminate between different severities of coronary stenosis and the number of major stenotic branches was assessed by calculating the area under the curve (AUC) using the receiver operating characteristic (ROC) curve, which distinguished between different severities of coronary stenosis and the number of major coronary stenotic branches by gender. The screening ability of each pulse pressure measure in differentiating the severity of coronary artery stenosis and the number of major coronary artery stenotic branches was evaluated.

## Results

### Basic characteristics of the study subjects

A total of 218 subjects were included in this study, with a median age of 62 years (range: 54–73). Among these, 130 subjects (59.9%) were male. Patients were divided into three groups based on their Gensini scores: T1 group (Gensini ≤ 3, n = 77), T2 group (3 < Gensini < 18, n = 68), and T3 group (Gensini ≥ 18, n = 73). The median Gensini score was 8 (range: 2 to 27.5). As the Gensini score increased, the following parameters also increased: age, CREA, GLU, D-Dimer, TG, TC, LDL, Non-HDL, LPP, RPP, LLPP, and RLPP. Conversely, HDL levels decreased. The differences in gender and smoking status among the three groups were statistically significant (P < 0.05; Table 1).

**Table 1:** Comparison of baseline data among the three patient groups.

Variables	T1(n = 77)	T2(n = 68)	T3(n = 73)	P-value
Male(%)	44(57.14)	34(50.00)	53(72.60)	0.019
Age	55(47-66)	67(58-73)	68(57-75)	< 0.001
Smoking(%)	21(27.27)	9(13.23)	21(28.77)	0.051
BMI	25.37 ± 4.46	23.98 ± 3.43	24.33 ± 3.69	0.082
Weekly exercise(%)	30(38.96)	18(26.47)	19(26.03)	0.15
Poor sleep quality(%)	28(36.36)	26(38.24)	18(24.66)	0.171
Cohabitant(%)	71(92.21)	59(86.76)	59(80.82)	0.122
Educational				0.326
Primary and below(%)	32(41.56)	37(54.41)	37(50.68)	
High school or junior college(%)	28(36.40)	16(23.5)	25(34.2)	
University and above(%)	17(22.1)	15(22.1)	11(15.1)	
BUN(mmol/L)	6.10(4.90-6.85)	5.80(5.03-6.78)	6.00(4.95-7.80)	0.472
CREA(umol/L)	69(56.50-82.78)	66.5(57.00-79.75)	73(62.50-89.50)	0.037
UA(umol/L)	335(281.50-385.50)	324.5(280.00-400.50)	354.00(296.00-424.50)	0.193
CYSC(mg/L)	1.04(0.90-1.26)	1.05(0.88-1.26)	1.08(0.89-1.31)	0.713
GLU(mmol/L)	5.12(4.47-6.09)	5.19(4.59-7.05)	5.78(4.97-8.12)	0.003
GSP(mmol/L)	1.69(1.46-1.87)	1.74(1.45-2.01)	1.66(1.37-1.84)	0.119
TG(mmol/L)	1.41(1.18-1.96)	1.53(1.11-2.40)	1.68(1.35-2.20)	0.035
TC(mmol/L)	4.16(3.44-4.76)	4.39(3.62-5.14)	4.52(3.82-4.98)	0.035
HDL(mmol/L)	1.09(0.92-1.30)	1.09(0.94-1.35)	1.03(0.85-1.16)	0.042
LDL(mmol/L)	2.14(1.58-2.68)	2.34(1.92-2.94)	2.49(1.96-2.87)	0.041
Non-HDL(mmol/L)	2.83(2.38-3.41)	3.34(2.47-4.02)	3.06(2.77-3.70)	0.009
ApoA1(g/L)	1.06(0.98-1.13)	1.04(0.94-1.12)	1.03(0.95-1.09)	0.226
ApoB(g/L)	0.69(0.55-0.88)	0.75(0.60-0.95)	0.71(0.54-0.89)	0.460
Lpa(mg/L)	181.30(95.04-298.27)	178.95(113.60-344.64)	202.52(113.60-408.82)	0.617
HCY(umol/L)	11.67(8.90-17.18)	13.52(9.72-18.37)	13.00(10.33-18.83)	0.414
D-Dimer(mg/L)	0.26(0.14-0.46)	0.38(0.19-0.64)	0.50(0.23-1.11)	< 0.001
LPP(mmHg)	38 (33 –42)	48 (43 –54)	61(54-72)	< 0.001
RPP(mmHg)	41(36.5-48)	49 (44 –55)	63(53-76.5)	< 0.001
LLPP(mmHg)	57(45.5-65)	64.5(59-72)	77(68-86.5)	< 0.001
RLPP(mmHg)	54 (47 –61)	67(58-73)	78(69.5-86)	< 0.001

*Note:* BUN: Blood Urea Nitrogen; CREA: Creatinine; UA: Uric Acid; CYSC: Cystatin C; GLU: Glucose;

GSP: Glycosylated Serum Protein; TG: Triglyceride; TC: Total Cholesterol; HDL: High-Density Lipoprotein; LDL: Low-Density Lipoprotein; Non-HDL: Non-High Density Lipoprotein Cholesterol;

ApoA1: Apolipoprotein A1; ApoB: Apolipoprotein B; Lp(a): ipoprotein (a); HCY: Homocysteine;

Dimer: D-dimer fibrin degradation product. LPP: left brachial artery pulse pressure；RPP: right brachial artery pulse pressure；LLPP: left ankle artery pulse pressure；RLPP: right ankle artery pulse pressure.

### Correlation analysis of right and left brachial and ankle arterial pulse pressure difference and cardiovascular influences with Gensini score

The results of the rank correlation analysis using Pearson and Spearman methods showed that gender, age, smoking, CREA, UA, GSP, TG, TC, HDL, non-HDL, LPP, RPP, LLPP, and RLPP were positively correlated with the Gensini score (P < 0.05); sleep quality was negatively correlated with the Gensini score were negatively correlated (P < 0.05; Table 2).

**Table 2:** Correlation analysis of PPs and sisk factors with Gensini score.

Variables	r	P-value
Sex	0.189	0.005
Age	0.176	0.009
Smoking	0.163	0.016
BMI	−0.008	0.912
Weekly exercise	−0.108	0.110
sleep quality	−0.153	0.027
cohabitant	−0.058	0.396
Education	0.047	0.493
BUN	0.039	0.563
CREA	0.134	0.043
UA	−0.143	0.034
CYSC	−0.014	0.836
GLU	0.080	0.237
GSP	−0.161	0.017
TG	0.184	0.006
TC	0.194	0.004
HDL	−0.158	0.020
LDL	0.152	0.025
Non-HDL	0.305	< 0.001
ApoA1	−0.096	0.160
ApoB	0.018	0.791
Lp(a)	−0.010	0.885
HCY	0.073	0.282
D-DImer	0.076	0.266
LPP	0.700	< 0.001
RPP	0.630	< 0.001
LLPP	0.461	< 0.001
RLPP	0.465	< 0.001

*Note:* BUN; CREA; UA; CYSC; GLU; GSP; TG; TC; HDL; LDL; Non-HDL; ApoA1; ApoB; Lp(a) and HCY:the same as Table 1. LPP；RPP；LLPP and RLPP: the same as Table 1.

### Association between the difference in pulse pressure between the right and left brachial and ankle arteries and Gensini score

**Fig 1:**
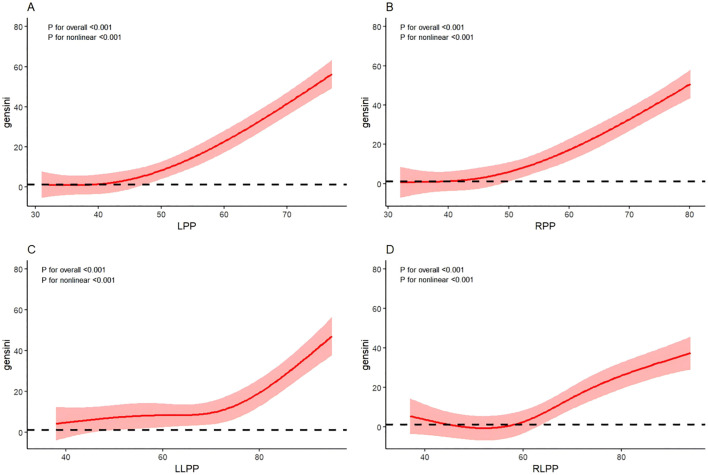
RCS analysis of the nonlinear association between PP measurements and Gensini score. *Note:* The X-axis represents continuous PP values(independent variables); the Y-axis represents the predicted Gensini score (dependent variable). A: left brachial PP (LPP), B: right brachial PP (RPP), C: left ankle PP (LLPP), D: right ankle PP (RLPP). Four knots were placed at the 5th, 35th, 65th, and 95th percentiles of PP distribution; solid lines indicate the estimated Gensini score, and shaded areas represent 95% confidence intervals.

Results of the multivariate logistic regression model analysis showed that LPP had a significant predictive effect on severe coronary stenosis (T3 group) both before and after adjustment for confounding factors (P < 0.001; Table 3). In Model 3, with the T3 group as the reference, the adjusted OR for LPP was 0.048 (95% CI: 0.010–0.227; P < 0.001) in the T1 group and 0.385 (95% CI: 0.187–0.792; P = 0.010) in the T2 group, indicating lower LPP was associated with a reduced risk of severe coronary stenosis in T1 and T2 groups compared with T3 group. However, there were no significant differences in RPP, LLPP, or RLPP among the groups (P > 0.05). Additionally, LPP had a significant predictive effect on the number of major coronary artery lesion branches. In Model 3, with the N2 group as the reference, the LPP value in the N0 group was 0.598 times that in the N2 group (OR: 0.598, 95% CI: 0.499–0.716; P < 0.001; see S1, Table a in S1 File); no differences were observed among the other groups. Multivariate-adjusted restricted cubic spline (RCS) analysis showed non‐linear trends between LPP, RPP, LLPP, RLPP, and the Gensini score, but the nonlinear patterns of association with the degree of coronary artery stenosis varied across different sites (Fig 1).

**Table 3:** Multivariate logistic regression analysis of PPs and categorical Gensini score tertiles in right and left brachial and ankle arteries.

	Model	T1 vs T3 (Reference: T3) OR (95%CI)	P-value	T2 vs T3 (Reference: T3) OR (95%CI)	P-value
LPP	1	0.265 (0.175-0.399)	< 0.001	0.613 (0.488-0.771)	< 0.001
	2	0.196 (0.109-0.354)	< 0.001	0.609 (0.481-0.771)	< 0.001
	3	0.048 (0.010-0.227)	< 0.001	0.385 (0.187-0.792)	0.010
RPP	1	0.863 (0.714-1.043)	0.127	0.893 (0.776-1.027)	0.893
	2	0.894 (0.722-1.108)	0.307	0.889 (0.771-1.024)	0.103
	3	0.904 (0.598-1.366)	0.631	0.873 (0.666-1.145)	0.326
LLPP	1	0.971 (0.856-1.100)	0.643	0.926 (0.850-1.009)	0.080
	2	0.979 (0.857-1.118)	0.753	0.929 (0.852-1.013)	0.095
	3	0.924 (0.719-1.192)	0.544	0.847 (0.699-1.028)	0.092
RLPP	1	1.019 (0.897-1.158)	0.773	1.037 (0.937-1.146)	0.484
	2	1.037 (0.904-1.189)	0.606	1.034 (0.930-1.150)	0.534
	3	1.105 (0.890-1.372)	0.367	1.077 (0.903-1.286)	0.409

*Note:* LPP, RPP, LLPP, RLPP: Same as Table 1; OR(95%CI): Odds Ratio(95% Confidence Interval)

Model 1: No adjustment for confounding factors;

Model 2: Adjustment for age and sex;

Model 3: Adjustments for BMI, smoking, CREA, GLU, TG, CHOL, HDL, LDL, non-HDL, and D-Dimer based on Model 2.

### Screening ability of right and left brachial and ankle artery pulse pressure difference for coronary artery stenosis

**Fig 2:**
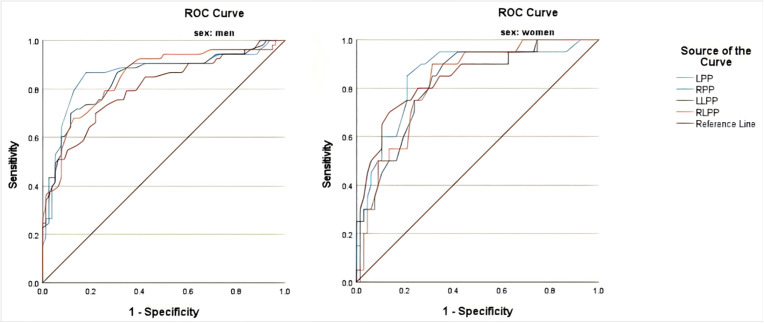
The ROC curve of PPs for predicting the degree of coronary artery stenosis in patients. *Note:* LPP, RPP, LLPP, RLPP: Same as Table 1.

The T1 and T2 groups were combined into a single group for analysis. The ROC curves for different genders were evaluated, revealing that the predictive values of LPP, RPP, LLPP, and RLPP for assessing the degree of coronary artery stenosis in males were 0.862, 0.842, 0.802, and 0.847, respectively (P < 0.001). The corresponding cut-off values were 51.5, 57.5, 69.5, and 73.5 mmHg. In females, the predictive values of LPP, RPP, LLPP, and RLPP for the degree of coronary artery stenosis were 0.854, 0.826, 0.842, and 0.823 mmHg, respectively (P < 0.001), with cut-off values of 51.5, 49, 72.5, and 66.5 mmHg (Fig 2, Table 4).

**Table 4:** Predictive value of PPs on the degree of coronary artery stenosis in patients by gender.

	Variables	AUC	Cut – off value	P-value	95%CI	Sensitivity (%)	Specificity (%)	Youden’s Index
Men	LPP	0.862	51.5	< 0.001	0.789-0.934	86.8	82.1	0.689
	RPP	0.842	57.5	< 0.001	0.769-0.915	69.8	88.5	0.583
	LLPP	0.802	69.5	< 0.001	0.723-0.881	69.8	78.2	0.480
	RLPP	0.847	73.5	< 0.001	0.777-0.917	67.9	87.2	0.551
Women	LPP	0.854	51.5	< 0.001	0.919-0.994	85	79.1	0.641
	RPP	0.826	49	< 0.001	0.835-0.972	90	64.2	0.542
	LLPP	0.842	72.5	< 0.001	0.740-0.944	70	86.6	0.566
	RLPP	0.823	66.5	< 0.001	0.730-0.917	90	68.7	0.587

*Note:* LPP, RPP, LLPP, RLPP: Same as Table 1.

In gender‐stratified ROC analysis for the number of major coronary artery lesions, AUC values were numerically higher for upper‐extremity pulse pressure measures than for lower‐extremity measures in both men and women. Specifically, LPP showed a numerically higher AUC than RPP in upper extremities (0.813 vs. 0.778), and RLPP showed a numerically higher AUC than LLPP in lower extremities (0.803 vs. 0.792; see S1, Fig a, Table b in S1 File).

## Discussion

Pulse pressure (PP) is recognized as a useful indicator in cardiovascular risk evaluation. PP is a direct indicator of arterial stiffness and is applicable to both treated and untreated hypertensive patients [29]. In the Framingham Heart Study, which included 2,040 participants, Haider et al. demonstrated that PP was associated with the risk of heart failure, regardless of age, over a mean follow-up period of 17.4 years [30]. Our results are consistent with previous studies, indicating that PP measured at bilateral brachial and ankle sites show independent associative value for identifying coronary stenosis. Nurses can use these findings in routine clinical screening to identify individuals at higher risk of coronary stenosis and provide timely referral for further cardiovascular evaluation.

Although multivariate logistic regression indicated that only LPP was statistically significantly associated with the Gensini score, pulse pressure values at the other three sites were higher in the T3 (high Gensini) group than in the T1 (low Gensini) group, yet without reaching statistical significance. This may be related to the relatively wide age range in the present study (since advancing age is closely linked to arterial stiffness [31]), regional differences in local hemodynamic characteristics and vascular compliance [32,33], as well as the inclusion of a mixed study population with and without coronary artery stenosis. Notably elevated PP may coexist with atherosclerotic burden, and previous studies suggested that elevated PP may be accompanied by inflammatory activation and reduced arterial wall elasticity [34]. This relationship emphasizes the importance of early detection and intervention. The results of the RCS indicated that the pulse pressure difference at various sites in the extremities is not simply linearly related to the degree of coronary stenosis; instead, it exhibits a complex nonlinear relationship. This finding aligns with the research by Nargesi AA and others [25], who demonstrated that the relationship between pulse pressure (PP) and coronary heart disease (CHD) follows a J-shaped curve.

Left brachial PP showed numerically higher AUC values in both men and women.In men, right ankle PP had numerically higher AUC; in women, left ankle PP was numerically higher. These sex-related differences may be explained by several factors. First, anatomical and physiological differences in arterial structure between men and women, including vascular diameter and wall elasticity, may influence pulse wave transmission to the lower extremities [35]. Second, previous studies have indicated that estrogen affects endothelial function and arterial compliance, which may alter the relationship between ankle pulse wave characteristics and coronary artery stenosis [36]. Even though left brachial PP showed relatively better numerical predictive performance, ankle PP also had favorable AUC values (all > 0.80), suggesting that ankle PP may also provide clinically useful information. The higher PP observed in the ankle may be related to distal pulse wave amplification, as reported in previous physiological studies [23]. From a clinical perspective, monitoring both brachial and ankle PP may offer complementary information for assessing arterial hemodynamics, although formal validation of combined use is warranted. However, we acknowledge that no formal statistical comparison of AUCs between different measurement sites was performed, and the potential added value of combined measurement needs to be verified in future studies with appropriate statistical methods. In this study, we also examined the predictive value of PPs for the number of stenotic branches. The findings were generally consistent with those for coronary stenosis severity, with left brachial PP showing numerically favorable AUC values. Sex-specific patterns were also observed for ankle PP, similar to the findings for Gensini score.

In the field of nursing, understanding the relationship between various physiologic parameters and disease states is critical to providing quality patient care. The positive correlation between brachial and ankle PP measurements and the degree of coronary artery stenosis, as well as with the number of stenotic branches in the main coronary artery, is particularly important in nursing. In nursing practice, if a nurse observes that a patient has persistently elevated PP at multiple sites, this finding may serve as a clinical cue to prompt further cardiovascular evaluation or referral. Importantly, the association between LPP and coronary artery stenosis remained significant after adjusting for multiple confounders, suggesting that brachial PP measurement could serve as a reliable and accessible screening tool across different patient populations. This is of value to nurses working in different healthcare settings, who can use these measurements to screen patients at risk for coronary artery stenosis. Furthermore, the non-linear relationship between PP and coronary stenosis identified in this study highlights the complexity of interpreting PP values in clinical practice. Nurses should be aware that patients with seemingly “normal” PP values may still be at elevated risk, particularly when other clinical factors are considered. This nuanced understanding can support more thoughtful clinical judgment and timely intervention. Medical workers should be aware of these differences when measuring blood pressure and use appropriate site-specific PP values to more accurately assess cardiovascular risk. By incorporating site-specific PP monitoring into routine practice, nursing staff may be better equipped to identify patients at higher risk for coronary stenosis in a timely manner, providing a foundation for individualized nursing interventions and improved cardiovascular care.

In a study comparing blood pressure measurements at the arm, calf, and ankle [37], the calf had the highest discomfort score, while the ankle had the lowest discomfort score. Therefore, blood pressure measurement at the ankle has significant advantages. When it is not desirable or possible to use the arm for measurement, the ankle should be the preferred alternative. Similarly, the measurement of ankle PP can provide nurses with additional important information, which helps to more comprehensively assess patients’ cardiovascular health status. This early detection capability is crucial for preventing cardiovascular events and improving patients’ long-term prognosis. Furthermore, monitoring ankle PP can also serve as a tool for nurses to dynamically assess patients’ cardiovascular status. During long-term care, nurses can regularly measure ankle PP, observe its trend over time, and evaluate the effectiveness of treatment and the progression of the disease. This dynamic monitoring helps nurses adjust treatment plans and ensure that patients receive the most appropriate care.

For ethical reasons, we did not stop the patient’s antihypertensive medication. Previous studies have shown that the relative increase in blood flow and vascular conductance in response to vasodilator stimulation is smaller in the legs compared with the forearms [38]. In our study, the relatively small number of patients receiving antihypertensive therapy, as well as the lower frequency and dose, may have influenced the results. Therefore, ankle PP may serve as a useful predictor, and its predictive value may vary by gender.

This information can help nurses prioritize patients for intensive care based on the number and extent of stenotic branches predicted by PP measurements.Future studies could allow for multimodal risk prediction, and combining PP with emerging biomarkers could improve the accuracy of multimodal risk prediction. Nurses can then use these more comprehensive risk assessment tools to provide more personalized care and early intervention for patients at risk of coronary stenosis.Although limited by a single-center design, these findings offer a practical, nurse-friendly screening tool for resource-limited settings.

This study had limitations. First, this was a retrospective cross-sectional study; thus, temporal relationships and causal inferences cannot be confirmed. Future prospective cohort studies are needed to verify the longitudinal predictive value of PP for coronary stenosis. Second, the sample size was relatively moderate, which may limit the generalizability of the findings. Third, although major confounders were adjusted in the regression model, residual confounding from unmeasured factors cannot be fully excluded. Fourth, the cardiovascular effects of blood glucose and glucose-lowering medications in diabetic patients have not been taken into account. Studies have shown that elevated glucose levels promote the formation of advanced glycosylation end products, which are associated with changes in the vascular wall and may affect the results of the study [39,40]. Fifth, while we observed sex-specific differences in predictive performance and numerically compared AUC values across sites, formal statistical comparisons of AUCs were not performed. Therefore, conclusions regarding the superiority of specific measurement sites or the added value of combined measurements should be interpreted with caution and require validation in future studies.

## Conclusion

In this study, left brachial pulse pressure (LPP) showed favorable predictive performance for severe coronary stenosis. Notably, gender differences were observed in the predictive efficacy of ankle pulse pressure: right ankle PP showed numerically higher AUC values in men, while left ankle PP showed numerically higher AUC values in women. These findings suggest that site-specific pulse pressure measurements selected according to gender may be useful for identifying patients at higher risk of coronary stenosis in clinical settings, although prospective studies are needed to validate their value for early screening, providing a practical and noninvasive tool for cardiovascular risk assessment in clinical nursing practice.

## Supporting information

S1 File Supplementary tables and figures. (DOCX)
