# Supplementary material for: Association of Brachial-Ankle pulse pressure with coronary artery stenosis severity: A sex-specific cross-sectional study in Chinese adults
Source: PLoS One. 2026 Jun 5;21(6):e0350269. doi: 10.1371/journal.pone.0350269 (PMC13240871; doi:10.1371/journal.pone.0350269)
Supplement: S1 File — (DOCX) [file pone.0350269.s001.docx]

**Table a.** Multivariate logistic regression analysis of pulse pressure in the right and left brachial and ankle arteries concerning the number of branches of major coronary artery vasculopathy.

|  | model | N0 vs N2 (Reference: N2)  OR (95%CI) | P-value | N1 vs N2 (Reference: N2)  OR (95%CI) | P-value |
| --- | --- | --- | --- | --- | --- |
| LPP | 1 | 0.576 (0.488-0.681) | < 0.001 | 0.810 (0.742-0.884) | < 0.001 |
|  | 2 | 0.582 (0.492-0.689) | < 0.001 | 0.801 (0.731-0.877) | < 0.001 |
|  | 3 | 0.598 (0.499-0.716) | < 0.001 | 1.210 (1.124-1.303) | < 0.001 |
| RPP | 1 | 1.031 (0.933-1.140) | 0.547 | 1.014 (0.947-1.086) | 0.688 |
|  | 2 | 1.019 (0.920-1.129) | 0.714 | 1.018 (0.950-1.090) | 0.616 |
|  | 3 | 1.050 (0.929-1.187) | 0.433 | 1.020 (0.943-1.103) | 0.620 |
| LLPP | 1 | 1.031 (0.954-1.113) | 0.442 | 1.020 (0.971-1.072) | 0.430 |
|  | 2 | 1.029 (0.952-1.112) | 0.468 | 1.020 (0.971-1.071) | 0.435 |
|  | 3 | 1.023 (0.935-1.120) | 0.617 | 1.017 (0.962-1.076) | 0.549 |
| RLPP | 1 | 0.958 (0.883-1.040) | 0.310 | 0.976 (0.927-1.027) | 0.351 |
|  | 2 | 0.962 (0.886-1.045) | 0.361 | 0.976 (0.927-1.028) | 0.360 |
|  | 3 | 0.955 (0.869-1.049) | 0.336 | 0.978 (0.922-1.037) | 0.452 |

*Note:* LPP, RPP, LLPP, RLPP: Same as Table 1.

Model 1: No adjustment for confounding factors;

Model 2: Adjustment for age and sex;

Model 3: Adjustments for BMI, smoking, CREA, GLU, TG, CHOL, HDL, LDL, non-HDL, and D-Dimer based on Model 2.

**Table b.** Predictive value of PPs for the umber of branches in major coronary vasculopathy by gender.

|  | Variables | AUC | **Cut - off value** | P-value | 95%CI | Sensitivity  (%) | Specificity  (%) | Youden's Index |
| --- | --- | --- | --- | --- | --- | --- | --- | --- |
| Men | LPP | 0.813 | 43.5 | < 0.001 | 0.736-0.891 | 86.8 | 72.7 | 0.595 |
|  | RPP | 0.773 | 48.5 | < 0.001 | 0.691-0.855 | 76.3 | 70.9 | 0.472 |
|  | LLPP | 0.748 | 68.5 | < 0.001 | 0.664-0.831 | 64.5 | 80 | 0.445 |
|  | RLPP | 0.803 | 66.5 | < 0.001 | 0.727-0.879 | 75 | 76.4 | 0.514 |
| Women | LPP | 0.778 | 44.5 | < 0.001 | 0.679-0.877 | 82.2 | 69 | 0.512 |
|  | RPP | 0.749 | 47.5 | < 0.001 | 0.646-0.853 | 75.6 | 69 | 0.446 |
|  | LLPP | 0.757 | 62.5 | < 0.001 | 0.656-0.858 | 75.6 | 69 | 0.446 |
|  | RLPP | 0.792 | 60 | < 0.001 | 0.693-0.890 | 82.2 | 71.4 | 0.536 |

*Note:*LPP, RPP, LLPP, RLPP: Same as Table 2.


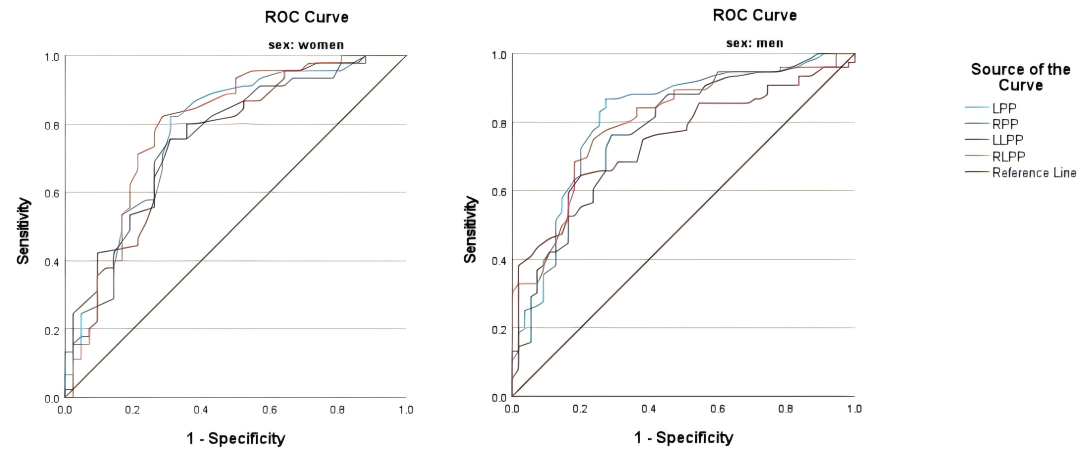


**Fig a.** The ROC curve of PPs for assessing the degree of coronary artery stenosis in patients.

*Note:* LPP, RPP, LLPP, RLPP: Same as Table 1.
